# Molecular Biomarkers of Epileptogenesis

**DOI:** 10.1007/s13311-014-0261-6

**Published:** 2014-02-25

**Authors:** Katarzyna Lukasiuk, Albert J. Becker

**Affiliations:** 1 The Nencki Institute of Experimental Biology, Polish Academy of Sciences, 3 Pasteur Street, 02 093 Warsaw, Poland; 2 Department of Neuropathology, University of Bonn Medical Center, Bonn, Germany

**Keywords:** Cerebrospinal fluid, epilepsy, plasma, seizures, traumatic brain injury

## Abstract

Epileptogenesis, a process leading to a reduced threshold for seizures after transient brain insults, as well as the mechanisms underlying the propensity to generate spontaneous epileptic seizures, are highly dynamic processes. Biomarkers—objective measures of biological processes—would be excellent tools for monitoring epileptogenesis and the dynamics of increased seizure propensity, as well as the potential to interfere, for example pharmacologically, with these key pathological aspects of epilepsy. Molecular biomarkers have revolutionized therapies, as well as response prediction and monitoring of therapies in other biomedical fields. However, high-impact molecular biomarkers are still not available in the context of epilepsy. Several factors, such as the large heterogeneity of epileptic syndromes and their underlying pathological patterns, as well as the limited availability of tissue samples, represent a particular challenge to the development of molecular biomarkers in epileptogenesis and epilepsy. However, substantial technical progress has been made recently with respect to biomarker characterization and monitoring by large throughput analysis on the genomic, mRNA, and proteomic levels, starting from minute amounts of brain tissue or body fluids, for example cerebrospinal fluid, blood, serum, or plasma. Given the substantial cellular- and network-level functional pathophysiology involved in epilepsy, it may be beneficial in the future to combine molecular analysis with other methods, such as imaging and electrophysiological biomarkers.

**Electronic supplementary material:**

The online version of this article (doi:10.1007/s13311-014-0261-6) contains supplementary material, which is available to authorized users.

## The Biomarker Concept

Biomarkers are defined as “measures” of disease processes, that is, factors that can be objectively determined and interpreted as indicators of pathogenic processes, such as those related to epileptogenesis and ictogenesis [1]. In the context of epileptogenesis, molecular biomarkers would be particularly helpful in identifying patients with an increased propensity of developing chronic spontaneous seizures after an epileptogenic insult. Furthermore, molecular biomarkers reflecting the presence, type, and severity of neuropathologically damaged tissue with epileptogenic potential should be of great value [2]. Considering the transition from epileptogenesis to a spontaneous epileptic condition and the semiological manifestation of an associated increased seizure propensity, molecular biomarkers that reflect dynamic changes in the seizure threshold will be of significant importance. It should be noted that potential molecular biomarkers for epilepsy development may also be suitable for monitoring or predicting therapy responses, for example by interventions retarding or even stopping epileptogenic processes, or raising the seizure threshold in the transition stage to chronic epilepsy, that is, the late stages of epilepsy development. Finally, biomarkers may become surrogate markers for spontaneous seizures, that is, measures used in therapeutic trials as a substitute for clinically meaningful end point, and through this eliminating the need to wait for spontaneous seizures to occur [2].

Epileptogenesis after a transient insult to the brain is accompanied by pathogenic processes that may serve as the source of potential biomarkers. These processes include reactive astrogliosis, the presence of activated microglia cells and leukocytes, blood–brain barrier (BBB) dysfunction, neuronal cell loss and neurogenesis, axonal regeneration, and—on the cellular level—altered expression and distribution of neurotransmitter receptors and ion channels [3, 4]. In the transition to the spontaneous seizure stage, the targeting of the receptors and channels may be particularly suited to modifying the epileptic threshold.

**Fig. 1:**
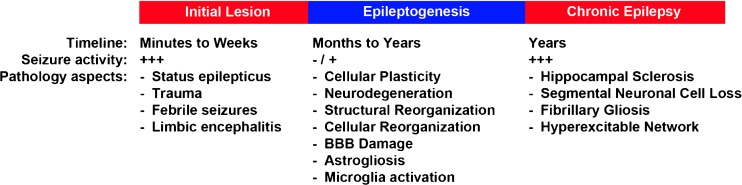
Schematic overview of key alterations during epileptogenesis. The key pathological elements of epileptogenesis are summarized. Epileptogenesis can emerge from different, potentially epileptogenic, insults. A plethora of mechanisms and potential biomarkers contribute to the conversion from a normal to a chronic epileptic brain structure. Notably, during epileptogenesis, occasional seizures can occur, but do not necessarily reflect the transition to the stage of chronic recurrent seizure activity (modified from [28]). BBB = blood–brain barrier

For the development of molecular biomarkers, focal epilepsies may represent a rather promising setting compared with other central nervous disorders, in which native disease tissue is rarely available. A significant number of patients with focal epilepsies develop pharmacoresistance to antiepileptic drugs. In many of these patients, surgical removal of the epileptogenic focus results in seizure control [2, 5]. The complementary availability of brain tissue from focal epilepsy patients undergoing surgery, and cerebrospinal fluid (CSF) and/or peripheral blood from different time points relative to the time point of neurosurgical intervention would be particularly valuable. If blood and CSF reflect pathogenic mechanisms in epileptogenic brain tissue, they would be well suited to the discovery of molecular biomarkers. A complementary approach may be the characterization and assessment of potential biomarkers in animal models of focal epilepsy and their potential translation to human patients (Fig. 1).

## Requirements of Biomarkers in the Epileptogenesis Context

In recent years, great progress has been made in using biomarkers to predict disease outcome, as well as therapy response in other central nervous system disorders, in particular in neuro-oncology [6]. This is particularly true for chemotherapy with procarbazine/CCNU/vincristine in patients with anaplastic oligodendrogliomas with 1p/19q co-deletion. Recent data from elderly patients with glioblastoma multiforme show the relevance of O(6)-methylguanine methyltransferase promoter methylation to the outcome of Temozolomid therapy.

However, developing biomarkers in the context of epilepsy is clearly substantially more complex. There are two main differences that we will consider here. First, in the context of potential epileptogenesis, after a transient insult to the brain, putative molecular biomarkers would need to be able to predict the future development of a disease that is not yet emergent at the time point of biomarker assessment. Second, and representing a major difference from neuro-oncology, molecular biomarkers for epilepsy will need to reflect the complex dynamics of seizure propensity. They could also constitute surrogate markers that demonstrate the potential to pharmacologically antagonize or retard epileptogenesis, thus avoiding epilepsy surgery for the patient. These considerations clearly show that although brain tissue from patients undergoing epilepsy surgery in the pharmaco-resistant stage should be used for the development of molecular biomarkers, such molecules may be particularly useful if they can be analyzed in the blood and/or CSF.

The field of neuro-oncology is somewhat ahead of the field of epileptogenesis in the consideration of blood-based biomarkers [7]. Similar to the function of molecular biomarkers for brain tumors, a transient dysfunction of the BBB may participate in the appearance of blood or serum biomarkers for epileptogenesis. In CSF, molecular correlates of cellular damage and inflammation associated with epileptogenesis or epileptic seizures, or the consequences of tissue damage and reorganization may be particularly pronounced if the affected anatomical structures are close to the ventricles. An ideal marker would be extremely sensitive and highly specific. However, the cellular composition of affected brain tissue, variability of blood and CSF marker half-life, and the dynamics of BBB integrity may substantially affect molecular biomarker levels in the blood and CSF. Similar to tumor markers, molecular biomarkers for epilepsy will not be perfect and perhaps do not need to be. It will be necessary to interpret molecular biomarkers applied to epilepsy with caution. Theoretically, several classes of molecules should be considered as potential molecular biomarkers, including cellular components derived from epileptogenic lesions, such as messenger RNAs, microRNAs, and alterations in DNA methylation reflecting pathology in the central nervous system. Proteins that reflect inflammation or neurodegeneration in an epileptic lesion should also be considered as potential molecular biomarkers. In the following discussion, we will present what has been discovered so far with respect to molecular biomarkers in the context of epileptogenesis and the transition to a spontaneous epileptic condition.

## Identification of Molecular Biomarkers for Epileptogenesis

Despite the growing knowledge of the molecular events occurring in the brain during epileptogenesis, there are currently no validated biomarkers that would allow the reliable prediction of increased likelihood of epilepsy development. Ideal biomarkers should not only be specific and sensitive, but also easily accessible. In the case of epileptogenesis, accessing the brain tissue of patients at risk is typically not feasible. Brain tissue is sometimes available from patients with traumatic brain injury (TBI) requiring surgical intervention, or from biopsies taken for diagnostic purposes, but such circumstances are rare, and direct evaluation of brain tissue is not included in the typical diagnostic process. Therefore, molecular biomarkers of epileptogenesis should either be based on brain imaging or derived from peripheral tissues.

As recently proposed by Engel et al. [2], the search for molecular biomarkers of epileptogenesis should start with proof-of-concept studies in a properly chosen animal model. Ideally, such a model should allow tissue sampling at different stages of epileptogenesis and a comparison between animals developing epilepsy to those that do not. The outcome measure should be occurrence of spontaneous seizures based on video electroencephalography monitoring. Next, a potential biomarker should be validated in different epilepsy models, and its sensitivity and specificity should be determined. Only then, the predictive value of the candidate biomarker should be tested in an appropriate group of patients for translation. There is no identified candidate biomarker that would fulfill the above described criteria yet.

Only a few reports in the literature propose candidate biomarkers together with outcome measures such as appearance of spontaneous seizures or their frequency. Although correlation with seizure frequency does not imply that a candidate molecule will become a biomarker, such studies are also included in this review as they may point to interesting mechanisms for epileptogenesis biomarker discovery. The majority of these studies were carried out based on experimental models of epilepsy. Despite the very preliminary state of this research, some ideas presented here may be promising and constitute the starting point for further studies.

A recent and intriguing approach complementary to the discovery of imaging biomarkers is the evaluation of brain metabolites associated with changes in brain metabolism during epileptogenesis. Glucose metabolism studies by positron emission tomography in both the pilocarpine and kainic acid models of epilepsy revealed hypometabolism in several brain areas during the latency phase and after the onset of recurrent seizures [8–10]. Interestingly, the level of hypometabolism in the entorhinal cortex has been shown to be correlated with the development of recurrent seizures [10]. Additionally, in the lateral fluid percussion model, glucose metabolism parameters detected in the ipsilateral hippocampus with positron emission tomography 1 week, 1 month, and 3 months after TBI were able to predict the development of epilepsy [11].

Metabolites other than glucose have been tested as potential imaging biomarkers. It has been shown that the level of myo-inositol, a metabolite linked to astrocyte activation, is elevated in the hippocampus during the latency phase in the pilocarpine and kainate models of epilepsy [12, 13], and that this increase can be detected with ^I^H-magnetic resonance spectroscopy. Although the level of myo-inositol reflects the extent of neuronal damage and neurodegeneration, it does not correlate with spontaneous recurrent seizures rat pilocarpine model [12]. Levels of glutathione, synthesized mainly in astrocytes, decrease early after status epilepticus (SE) but subsequently increase gradually [12, 14]. Interestingly, glutathione levels in the hippocampus, measured with ^I^H-MRS during epileptogenesis, correlate negatively with neuronal cell loss and with the frequency of recurrent seizures observed in the chronic epileptic stage [12]. In animal models, levels of lactate transiently increase during epileptogenesis; however, there is no correlation with the frequency of seizures later on [12, 15]. The level of another brain cell-derived metabolite, N-acetyl-aspartate, is decreased early following SE and in the latency phase, possibly reflecting neuronal loss and/or changed neuronal metabolism [12, 15].

The fact that blood, serum, and plasma are easily accessible makes them very attractive sources of biomarkers. To date, only levels of inflammatory proteins in the circulating blood have been proposed as potential markers of epileptogenesis and have been studied in experimental insult models. The rationale was that their presence in blood may result from either “spillover” of cerebral inflammatory molecules or even cells, or be due to peripheral inflammation. Plasma levels of inflammatory proteins C-reactive protein, interleukin 1-beta, and interleukin 6 were studied in the angular bundle stimulation model of the temporal lobe epilepsy, but no change was detected acutely after SE, neither during the latency phase nor in chronic epilepsy [16].

## Potential Biomarkers in TBI

TBI may result in the development of epilepsy. Although there is no approved biomarker of TBI in clinical use, several candidate molecular biomarkers exist. These have been evaluated at acute insult stages to examine their correlation with the level of damage and to predict functional outcomes in terms of mortality, secondary pathologies, and neurological parameters (reviewed in [1, 17, 18]).

Candidate biomarkers of TBI include serum or CSF levels of protein leaking from the brain, which may represent a distinct correlate of brain damage. Intriguing candidates for such biomarkers, detected in trauma patients, as well as in animal models, are S100 calcium binding protein B (S100B), neuron-specific enolase, glial fibrillary acidic protein, ubiquitin carboxyl-terminal hydrolase L1, myelin basic protein, and tau [1, 17, 18]. In addition to dynamic protein concentrations, levels of metabolites, including cyclic adenosine monophosphate, lactate, pyruvate, glycerol, glutamate, norepinephrine, homovanillic acid, hydroxyindolacetic acid, and N-acetylaspartate, as well as products of lipid peroxidation, such as F2-isoprostane, are altered after TBI and correlate with injury severity [1, 17]. Current efforts to identify new candidate protein biomarkers in the CSF of TBI patients apply proteomics methods [19].

In the studies described above, candidate TBI biomarkers that would include the development of epilepsy as an outcome measure are lacking.

## Potential Biomarkers in SE

SE is a risk factor for epilepsy development in humans. The majority of animal models of epileptogenesis use chemically- or electrically-induced SE as an epileptogenic insult. Molecular changes in the brain induced by SE are well described [3, 20], and several peripheral biomarkers have already been suggested (Table 1).

**Table 1:** Summary of potential biomarkers for epileptogenesis in cerebrospinal fluid (CSF) and blood serum

Molecule	Biomaterial	Remarks [reference]
S100B	Serum/CSF	Levels correlate with injury severity after trauma and have predictive value on neurological outcome [1, 17, 18]
NSE	Serum	Levels correlate with neurologic outcome after trauma and reflect neuronal damage after SE [1, 17, 18, 23, 24]
GFAP	Serum/CSF	Levels correlate with injury severity after trauma and have predictive value on neurologic outcome; increase in CSF correlates with epileptogenesis stage in the kainic acid SE model [1, 17, 18, 22]
UCHL1	CSF	Increase in CSF correlates with epileptogenesis in the kainic acid model [22]
MBP	Serum	Released in the course of brain trauma; predictive value for epileptogenesis to be determined [1, 17, 18]
Tau	Serum/CSF	Released in the course of brain trauma; predictive value for epileptogenesis to be determined [1, 17, 18]
miR-9	Serum/brain	Increased after traumatic injury; predictive value for epileptogenesis to be determined [20]
Prolactin	CSF, serum	Transiently increased in serum of SE animal model in early epileptogenesis; serum levels in SE patients not increased; predictive value for epileptogenesis to be determined [25, 26]

S100B = S100 calcium binding protein B; NSE = neuron-specific enolase; GFAP = glial fibrillary acidic protein; UCHL1 = ubiquitin carboxyl-terminal hydrolase L1; MBP = myelin basic protein; miR-9 = microRNA-9; SE = status epilepticus

The CSF levels of several proteins, including glial fibrillary acidic protein and ubiquitin carboxyl-terminal hydrolase L1, have been shown to be elevated acutely after SE [21]. An SE-induced increase in the serum level of neuron-specific enolase has been observed in humans and in animal models [22, 23]. The level of prolactin in plasma and serum has also been shown to transiently increase after SE in animals and humans [24, 25].

Profiling of the blood transcriptome early after kainic acid-induced SE in rats has shown that the levels of several messenger RNAs are dynamically affected [26]. Additionally, changes in blood microRNA profiles were found after kainic acid-induced SE in rats [27]. These changes, however, have not been validated, and their functional implications for the development of epilepsy are not clear.

The prognostic value of these biomarkers for epilepsy development has not been studied. Moreover, the specificity of some of these changes for SE is questionable because similar alterations may also occur after seizures or TBI.

## Conclusions and Perspectives

### Pattern Combination

Several features of epileptogenesis hamper the discovery of biomarkers. First, epileptogenesis is a dynamic process. Several measures—imaging, and histological and molecular data—indicate that pathological changes occur in the brain sequentially and in parallel, including neurodegeneration, inflammation, and functional and structural neuronal plasticity. Time lines for each of these phenomena overlap and may differ depending on etiology, environmental factors, or patient genetic susceptibility. As different mechanisms predominate at distinct phases of epileptogenesis, different biomarkers will presumably be necessary to predict the development of seizures over the course of epileptogenesis. Furthermore, an important issue in acquired epilepsies will be the specificity of the candidate biomarker. Particularly frequent causes of epilepsy, including traffic accidents and military injuries, often involve multi-organ injuries. It will be challenging to identify biomarkers with enough specificity to identify ongoing epileptogenesis in such cases.

Taking into account the complexity of epileptogenesis, potential panels of comprehensive, complementary biomarkers (imaging, electroencephalography, molecular) evaluated at different stages (i.e., different time points after the precipitating injury) may be required to provide reasonable predictive value.

### Translation of Biomarkers Into Therapeutic Approaches

Given the basic concept of biomarkers in epileptogenesis and the decreased seizure threshold prediction at the transition from epileptogenesis to the chronic seizure stage, it may be expected that molecular biomarkers that are either up- or down-regulated, or altered, for example by phosphorylation, at this stage not only correlate with these features, but also play a putative functional role in these processes. It would be ideal to detect biomarkers that correlate with specific pathologic aspects of epilepsy as they may also give insights into therapeutic options. New avenues for epilepsy biomarker discovery and therapy development may result from studies on the manipulation of gene expression in animal models of insult-induced epileptogenesis employing, for example, small interfering RNA-mediated antagonism of abundant gene transcription in downstream pathways or the compensatory overexpression of certain molecules, which are reduced in epilepsy.

## Electronic supplementary material

Below is the link to the electronic supplementary material.

ESM 1 (PDF 1225 kb)
